# Isolation and Molecular Identification of *Mycobacterium bovis* from Slaughtered Cattle in Nekemte Municipality Abattoir, Ethiopia

**DOI:** 10.1155/2023/9911836

**Published:** 2023-12-16

**Authors:** Tola Mezgebu Gemeda, Eyob Hirpa Tola, Balako Gumi Donde, Muse Girma Abdela, Hika Waktole Ayana

**Affiliations:** ^1^School of Veterinary Medicine, Wollega University, Nekemte, Ethiopia; ^2^College of Veterinary Medicine and Agriculture, Addis Ababa University, Bishoftu, Ethiopia; ^3^Addis Ababa University, Aklilu Lemma Institute of Pathobiology, Addis Ababa, Ethiopia

## Abstract

Tuberculosis (TB) is a zoonotic disease that can spread from animals to humans as well as from human to human. Little research has been conducted on bovine tuberculosis prevalence and molecular characterization in the western part of Ethiopia. To investigate this, a cross-sectional study was conducted on slaughtered cattle at the Nekemte municipal abattoir between January 2020 and June 2021. A detailed postmortem examination, culture, acid-fast staining technique, molecular characterization using RD4 deletion, and spoligotyping were all carried out. Based on a detailed postmortem examination, the overall prevalence of bovine tuberculosis was 7.8% (80 of 1020). Mycobacterium isolation confirmed only 12.5% (10/80) of the suspected tuberculosis tissue lesions. With acid-fast bacilli staining, all Mycobacterium spp. isolates (*n* = 10) were positive. However, only 9/10 isolates were confirmed to be *M. bovis* with RD4 molecular deletion typing. Spoligotyping revealed that 55.6% (5/9) of the isolate patterns had previously been reported, but 44.4% (4/9) of the isolates were new. In the current investigation, it was discovered that 80% (4/5) of the *M. bovis* strains circulating in the cattle population of study regions were SB2233 (2/5) and SB0134 (2/5), whereas 20% (1/5) of the strains corresponded to SB1176, which is compatible with previously documented *M. bovis* spoligotypes. These findings suggested that *M. bovis* was the main cause of bovine tuberculosis in the study area and posed a risk of disease transmission from cattle to humans due to low levels of public health awareness. As such, improved awareness among citizens and the development of control policies are warranted.

## 1. Introduction

Tuberculosis (TB) is caused by Mycobacterium tuberculosis complex (MTBC). MTBC constitutes a significantly genetically similar group of bacteria that cause tuberculosis in a wide range of hosts. They are rod-shaped, acid-base fast, aerobic, slow-growing intracellular pathogens that destroy phagosomal cells to maintain and evade the immune system [1]. TB is the 13th leading cause of death and the second leading infectious killer after COVID-19 (above HIV and AIDS) [2]. It is a severe and a recurrent disease that poses a significant health risk to humans and animals [3, 4]. It is listed as a reportable disease in the World Organization for Animal Health Terrestrial Health Code [5].

*Mycobacterium bovis* causes bovine tuberculosis [6]. It still continues to cause a significant impact on livestock productivity and on livelihoods of communities [7]. Indirect transmission might be facilitated if MTBC bacteria persist in the environment long enough to represent a risk of exposure to different species sharing the same habitat [8]. The only policy that has led to effective eradication of bovine tuberculosis has been the test and slaughter policy [6]. Bovine tuberculosis has been reported from January 2017 to June 2018, and of the 188 countries and territories reporting their BTB situation to the OIE, 82 countries (44%) were affected African countries [9, 10]. Among the 82 affected countries, 29 (35.4%) countries reported the presence of BTB in both livestock and wildlife. Two (2.4%) countries reported BTB present only in wildlife, compared to 51 (62.2%), which indicated that only livestock were affected. The majority (62%) of the affected countries had implemented some of the relevant measures. However, 3% of them had not applied any of these measures and, therefore, required strengthened control efforts [10].

Bovine tuberculosis is an endemic disease of cattle in Ethiopia, according to previous tuberculin testing surveys and meat inspections of abattoirs [11]. The prevalence in intensive dairy herds varied from 22 to 47% [12], with a prevalence of 1.1%–24.7% in abattoirs and 3.5–50% in crossbreed farms [13–15]. Unlike human TB, it has not received more focus on research, and several factors impair disease control and eradication, along with the limited sensitivity of diagnostic tests and slaughter control strategies not applied due to economic constraints, but their effectiveness is applicable in developed countries [16]. Due to inadequate comprehensive abattoir surveillance and a lack of diagnostic facilities, the BTB has limited information [10, 17], particularly on the genotypic characteristics of *M. bovis*, a strain affecting the cattle population in Ethiopia [18]. The research aims and objectives of the study were to determine the prevalence of bovine tuberculosis in cattle slaughtered at the Nekemte municipality abattoir and to identify the *M. bovis* strains through molecular-based techniques including RD4 gene deletion and spoligotyping [19–21].

## 2. Materials and Methods

### 2.1. Study Area

**Figure 1:**
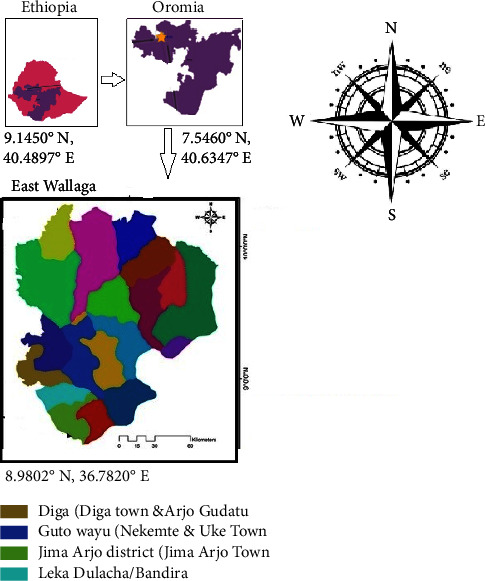
Map of the study area (Nekemte Town, Oromia, Ethiopia).

The town of Nekemte is located in the East Wollega zone, western Oromia, Ethiopia. Nekemte is 328 kilometers from Addis Ababa. Nekemte has a single municipal abattoir. The town is situated at 9 5 N, 36 33 E and is 2088 meters above sea level. The total number of cattle population in the East Wollega zone was 228, 2100. Nekemte is the 14^th^ most populous town in Ethiopia, with an estimated population of 110,688.  Figure 1 depicts a map of the study area.

### 2.2. Study Animals

A total of 1020 apparently healthy adult cattle were slaughtered in the abattoirs located within the Nekemte municipality. The cattle used for this study mainly originated from different districts of the East Wollega zone, such as Jima Arjo, Leka Dulacha (Bandra), Diga (Diga and Arjo Gudatu town), and Guto Wayu (Nekemte and Uke). Basic animal information, including sex, age, breed, body condition score, and market, was collected and recorded during the antemortem examination. All cattle included in the study were managed under extensive farm management systems and were not tested for tuberculosis. All apparently healthy cattle that passed the antemortem examination and were fit for slaughter were included in this study.

### 2.3. Study Design

A cross-sectional study design was conducted from September 2020 to October 2021 to assess the prevalence of bovine tuberculosis, identify the circulating *Mycobacterium bovis* in the area and assess the knowledge, attitude, and practice of the community towards bovine tuberculosis.

### 2.4. Sample Size Determination

During the study period, all animals coming at abattoir from various markets were considered for sample. The Thrusfield [22] formula was used to calculate the sample size. The previous work of Woyyesa et al. [23] used the expected prevalence (Pexp) which was 5.9%, and the 95% confidence interval (CI) with 5% accuracy and sample size was determined as follows:

(1) $$\begin{array}{c} N=\frac{1.96^{2}\times Pexp\left(1-\mathrm{Pexp}\right)}{d^{2}}\\ =\frac{1.96^{2}\times 0.059\left(1-0.059\right)}{0.0025}=85,\\ N=\text{districts sample}*2*\text{ number of }d\text{istrict}s\\ =85*2*6=1020, \end{array}$$

where *N* is the required sample size, Pexp represents the expected prevalence, and *d* represents the desired absolute accuracy (5%). As a consequence, the sample size for bovine TB lesions was determined for each district to be 85. The sample size was increased twice in each to improve precision and limit the potential of errors. As a result, the study's overall sample size for six districts was 1020.

### 2.5. Diagnostic Tools

**Figure 2:**
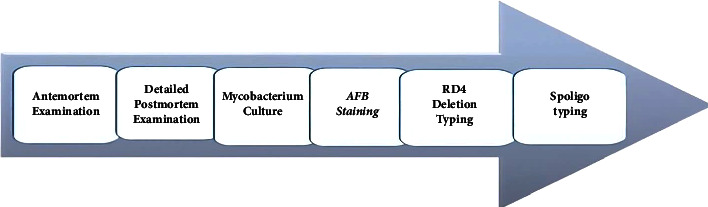
List of investigation techniques utilized in the study.

An antemortem inspection, detailed postmortem investigation, acid-fast bacilli (AFB) staining, culture, and molecular characterization techniques such as RD4 deletion and *M. bovis* spoligotyping tools were used. The types and sequence of diagnostic tests performed in the investigation are depicted in Figure 2.

### 2.6. Antemortem Examination Technique

Each tested animal was examined for clinical sign before being slaughtered. During the antemortem examination, all vital signs were checked, and other details such as each animal's sex, breed, origin, and body condition score were recorded. All animals were not received a skin test for bovine tuberculosis. The body condition score for each study animal was determined using Jacob Segers' guidelines [24]. As a result, the study animals were classified as thin (scoring 1–3), borderline (scoring 4), medium-sized (scoring 5–7), or obese (scoring 8–9). An age estimate was made according to Torell and collaborators [25]. Since every animal killed throughout the study period was older than five years, they were all classified as adults (Tables 1 and 2).

### 2.7. Isolation of *Mycobacterium bovis* from Tuberculosis Lesions

The OIE and the Ministry of Agriculture's Department of Meat Inspection and Quarantine techniques were employed for the detailed postmortem examination [26, 27]. All lymph nodes, livers, kidneys, and lungs were visualized, palpated, and incised to a size of 2 mm with stainless surgical blades to facilitate in the detection of TB lesions from each animal. Abscesses, caseous masses, and tubercles were studied under bright light on the cut surfaces of tissues in isolated locations [27]. The animal was classified as suspected tuberculosis lesion when tuberculosis lesion was found, and if not as nonlesioned.

All suspected tuberculosis lesion tissue samples were collected using independent sterilized disposable gloves, surgical blades, and universal bottles to avoid cross contamination [28]. The specimens were transported to the Wollega University Veterinary Laboratory for further examination, followed by transport to Akililu Lema Institute of Pathobiology (ALIPB) at Addis Ababa University for *Mycobacterium* sp. isolation. Finally, the samples were stored at −20°C until bacteriological culture and acid-fast bacilli staining was performed (Appendix).

The study used duplicate egg-based Löwenstein–Jensen medium supplemented with glycerol or pyruvate to culture mycobacteria, following standard procedures described in WHO [29] and Biffa et al.'s [30] study. The medium with glycerol was used to favor the growth of *M. tuberculosis*, while the medium with pyruvate was used to favor the growth of *M. bovis*. The slants were incubated at 37°C and observed daily for colony growth during the first week and then weekly thereafter. If no growth was observed on the slants by week 8, they were considered negative.

### 2.8. Region of Difference (RD4) Deletion Typing

Two loopfuls of whole colonies from each isolate were harvested for RD4 deletion typing. Colonies were mixed in 200 *μ*L of Tris-borate-EDTA (TBE) buffer, heat-killed at 80°C for 1 hour using a heat block, and stored at 80°C until molecular detection was performed. The isolates were confirmed as *M. bovis* by deletion typing of the RD4 region using a previously described PCR protocol [19]. RD4 deletion typing, a chromosomal deletion characteristic of *M. bovis*, was performed by conventional PCR. RD4 internal forward primer (RD4 intF): 5′-ACA CGC TGG CGA AGT ATA GC-3′, RD4 flanking forward primer (RD4flankF): 5′-CTC GTC GAA GGC CAC TAA AG-3′, and RD4 flanking reverse primer (RD4flankR): 5′-AAG GCG AAC AGA TTC AGC AT-3′ were used to check for the presence of the RD4 locus. Each PCR consisted of 7.1 *μ*L RNase- and DNase-free water (Qiagen), 10 *μ*L Hot Start Taq Master mix, 2.1 *μ*L heat-killed mycobacterial DNA template, and 0.3 *μ*L of each primer, for a total volume of 20 *μ*L. *M. tuberculosis* H37Rv and *M. bovis* 2122/97 were used as positive controls while distilled water was used as negative control. The mixture underwent thermal cycling with an initial denaturation at 96°C for 15 min followed by 35 cycles of 96°C for 1 min, 55°C for 1 min, and 72°C for 1 min and a final elongation at 72°C for 10 min. PCR products were electrophoresed on a 1.5% agarose gel in 1X TBE running buffer with SYBR Safe dye at a 1 : 10 ratio and a 100 bp DNA ladder. Gels were visualized using a Benchtop 2UV transilluminator. Presence of the RD4 locus (*M. tuberculosis* and *M. africanum*) gives a 335 bp product (RD4 intF + RD4flankR) while its absence (*M. bovis*) gives a 446 bp product (RD4flankF + RD4flankR).

### 2.9. Conventional Spoligotyping

Spoligotyping was carried out according to the previously described method by Kamerbeek et al. [31], and a noncommercial spoligotyping membrane manufactured by the Animal and Plant Health Agency (UK) was used for hybridization and spacer detection. The presence or absence of spacer signals to identify the spoligotype was used. The reference strains *M. tuberculosis* H37Rv and *M. bovis* AF2122/97 were used as controls. Spoligotype bovis (SB) numbers were identified by comparing generated spoligotype patterns to the *M. bovis* spoligotyping database (https://www.mbovis.org/).

### 2.10. Questionnaire Survey

A face-to-face interview was conducted using a semistructured pretested and translated (from English to Afaan Oromo) questionnaire. Predefined questions are presented in a standardized questionnaire containing nine knowledge questions, attitude questions, and practice questions that enable access to quantitative and qualitative data related to bovine tuberculosis control and prevention. The survey sample size was calculated using the formula detailed by Arsham [32]:

(2) $$\begin{array}{c} n=\frac{0.25}{\text{SE}2}\\ =\frac{0.25}{0.05^{2}}+20\%=120, \end{array}$$

where *n* = number of population and SE = standard error

The study utilized a multistage random sampling technique to select respondents in Nekemte town. In the first stage, six kebeles were randomly selected out of the twelve total kebeles. In the second stage, twenty household heads were randomly selected from each of the six kebeles, for a total sample of 120 respondents. Eligible respondents were permanent residents of Nekemte for over six months and were financially responsible for their households. If a selected household did not contain an eligible respondent, the survey team moved to the next available household until reaching the target sample size. This multistage approach allowed for a representative sample of Nekemte household heads to be surveyed.

### 2.11. Data Management and Analysis

All data were entered into Microsoft Excel and then imported into SPSS version 25 for analysis. Descriptive statistics including frequencies and percentages were calculated for the prevalence of gross pathological lesions. Prevalence was calculated as the proportion of cattle with TB lesions out of the total cattle examined. Frequencies were used to summarize pathological scoring data. Variation between different factors was analyzed using chi-square tests. Odds ratios with 95% confidence intervals were calculated to assess the strength of associations. *P* values less than 0.05 were considered statistically significant.

### 2.12. Ethical Clearance

Ethics in animal handling has been evaluated and approved by the Wollega University School of Veterinary Medicine Ethical Approval Committee against international and national guidelines for the humane treatment of animals and conforms to relevant legislation. The Wollega University Research Ethics Committee approved the research proposal “Isolation and Molecular Detection of *Mycobacterium* from Cattle Slaughtered in the Municipal Abattoir of Nekemte Town, Ethiopia” on 1/7/2020 under approval code: WUSVM/7/2020.

## 3. Results

### 3.1. Prevalence of Bovine Tuberculosis Using Detailed Postmortem Examination

The study examined a total of 1,020 cattle slaughtered in Nekemte, Ethiopia, between September 2020 and October 2021. Various lymph nodes and organs were inspected for gross lesions indicative of bovine tuberculosis. The retropharyngeal lymph nodes in the head region were examined, with 8 out of 1,020 (0.78%) cattle testing positive. In the thoracic region, the bronchial lymph nodes were positive in 17 out of 1,020 cattle (1.7%), while the mediastinal lymph nodes had a higher positivity rate of 38/1,020 (3.7%). Lung lesions were found in 5 out of 1,020 cattle (0.5%). In the abdominal region, 10 out of 1,020 cattle (1.0%) had positive mesenteric lymph nodes.

Overall, gross tuberculous lesions were detected in 80 of the 1,020 cattle screened, yielding an observed bovine tuberculosis prevalence of 7.8% in this sample. The mediastinal lymph nodes in the thorax demonstrated the highest level of positivity. These results provide evidence that bovine tuberculosis is present and circulating among cattle slaughtered in the Nekemte area of Ethiopia. The distribution of TB lesions in different organs and tissues concerning the anatomical site is presented in Table 3.

The study found 8.8% of female and 7.65% of male cattle tested positive for TB gross lesions, though this difference was not statistically significant (*p* = 0.260). Cattle with medium body condition scores had a higher positivity rate (8.87%) compared to those with good scores (2.08%), but this only approached significance (*p* = 0.082). In contrast, crossbred cattle showed a significantly higher positivity rate (17.6%) than local breeds (7.6%) (*p* = 0.009), indicating greater risk. Positivity varied by origin from 3.5% (Diga) to 13.0% (Nekemte), though differences were not significant (*p* = 0.192). In summary, breed was the only factor with a statistically significant association, as crossbreds appeared at higher TB risk than local breeds. Other factors such as sex, body condition, and origin did not have significant relationships with lesion prevalence, though more research could further analyze potential risk factors.  Table 4 indicates the prevalence of bovine tuberculosis among different groups by gender, body condition, and breed.

### 3.2. Culture and Acid-Fast Bacilli Staining

Mycobacterial growth was observed in 10 of 80 (12.5%) tissue samples following culture inoculation. Growth occurred in 9 samples (11.3%) on the pyruvate-enriched Lowenstein–Jensen (LJ) medium and 1 sample (1.3%) on the glycerol-enriched LJ medium. Colonies appeared smooth and white, consistent with mycobacterial morphology. Acid-fast staining of the 10 culture-positive isolates revealed acid-fast bacilli with coccoid, single rod, and clumped forms in 9 isolates. The remaining 1 isolate stained negative for acid-fast bacilli.

### 3.3. Molecular Characterization of Mycobacteria Isolates

#### 3.3.1. The RD4 Deletion Techniques

**Figure 3:**
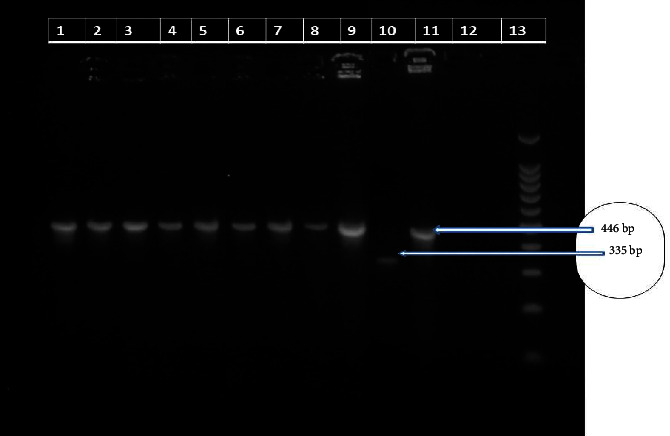
Gel electrophoresis separation of PCR products by RD4 deletion typing lanes 1, 2, 3, 4, 5, 6, 7, 8, and 9 were positive for *M. bovis*; lane 10: *M. tuberculosis* H37Rv (positive control, 335 bp); lane 11: *M. bovis* 2122/97 (positive control, 446 bp); lane 12 was distilled water (negative control); and lane 13 was a molecular ladder (100 bp).

PCR targeting the RD4 deletion region was performed on the 9 AFB-positive isolates to differentiate *Mycobacterium bovis.* All 9 isolates generated a 446 bp PCR product, indicative of RD4 deletion and consistent with *M. bovis*. Gel electrophoresis confirmed the presence of the 446 bp band for all isolates (Figure 3). The RD4 deletion is a distinguishing feature of *M. bovis* strains.

#### 3.3.2. Spoligotyping of *Mycobacterium bovis* Isolates

**Figure 4:**
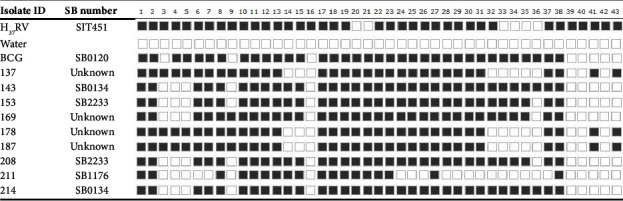
Spoligotype result of the 9 isolates.

Spoligotyping of the nine *M. bovis* isolates, which were positive for RD4 deletion, showed that five out of the nine samples had a typical *M. bovis* signature of spoligotyping, with spacers 3, 9, 16, and 39–43 being absent. These identified spoligotypes revealed unique patterns that distinguished the samples from other members of the Mycobacterium tuberculosis complex (MTBC). After being compared against the global spoligotype database (https://www.mbovis.org) [33], three distinct patterns were observed among these five isolates: SB0134, SB1176, and SB2233. However, results for the remaining four isolates were unknown as their spoligotypes did not match those available in the database. Among the five identified patterns, spoligotype SB2233 (two isolates) and SB0134 (two isolates) were the dominant strains, followed by SB1176 (one isolate). The four remaining isolates had spoligotypes not identified within the international spoligotyping database, suggesting they were unknown strains. Among the unidentified isolates, isolate ID169 had a pattern somewhat similar to SB2233 except for the presence of spacer 9 in isolate ID169. The presence of spacer 9 is likely an artifact in the isolate ID169 spoligotype. In addition, the other three unidentified isolates 137, 178, and 187 share a similar pattern, lacking spacers 14–16, 32–36, 39, 40, and 42. In conclusion, *M. bovis* is characterized by the absence of spacers 3, 9, 16, and 39–43. However, the presence of spacers 3, 9, and 16 as well as 41 and 43 in the three unknown isolates 137, 178, and 187 indicates they are not *M. bovis* strains. The spoligotype results of these nine isolates are displayed in Figure 4.

## 4. Questionnaire Survey

The study included 120 participants, with 63% being male and 37% being female. Out of the total participants, 39% were young aged between 18 and 29 years and 61% were adults aged between 30 and 65 years. In terms of education, 15% of participants had no education, 34% had completed primary education, and 51% had completed secondary education. Bovine tuberculosis (BTB) is an infectious disease mainly found in cattle. It can also spread to humans and other animals. Assessing public awareness about this zoonotic illness is vital for enhancing prevention and control. However, a recent survey of 120 participants revealed major knowledge gaps regarding BTB. Only 26.7% of the respondents had prior understanding of bovine TB, while the vast majorities (74.3%) were unaware of the disease's existence. In addition, merely 23.3% knew that BTB can transmit from cattle to people. Yet, a substantial 76.7% did not know about the zoonotic capacity of bovine tuberculosis. Respondents' attitudes towards consuming raw milk and raw meat revealed that 14.1% of the participants had the habit of consuming raw milk and 68.2% reported a high habit of consuming raw meat. The study found low tuberculosis prevention practices among the participants. Only 22.5% of the participants knew tuberculosis prevention practice, and 81.5% of them did not know appropriate prevention practices.

## 5. Discussion

The overall prevalence of bovine tuberculosis based on gross lesions was found to be 7.8%. The present finding agrees with the previous report in other parts of Ethiopia, such as Gimbi (7.75%), Gonder (8.6%), and areas around Gimbi town (7.9%), northwestern Ethiopia (7.1%), Wuchale-Jida district (6.79%), Adama municipal abattoir (7.96%), and Hawasa municipal abattoir (7.95%) as reported in earlier studies [31, 34–39].

The current finding shows a moderate increase compared to previous studies conducted at Nekemte municipal abattoir (5.9%) [23], Hawasa municipal abattoir (5.84%) [13], Bishoftu municipal abattoir (5.7%) [40], and Jima municipal abattoir (5.4%) [41]. However, it is still lower than the findings from Gambela municipal abattoir, which recorded 13.2% [18], and Gondar Elfora abattoir with 11.7% [42] and 15.9% [11]. Previous studies conducted at Nekemte municipal abattoir have indicated a different prevalence of bovine tuberculosis compared to the findings obtained in the current study. These differences can likely be attributed to various factors, such as variations in the origin and age of slaughtered animals, housing systems for these animals, as well as infection rates across distinct sites. It is possible that the different sources of cattle taken to the abattoir and their body condition resulting from distinct production systems and breeds can alter the epidemiology of tuberculosis within an animal population [13, 14, 43].

In this study, out of 80 tissue samples subjected to LJ medium cultures with added pyruvate and glycerol supplements, only 10 tissues showed growth of visible smooth and white colony.

Out of the 80 tissue samples, tissue growth was observed in 10 (11.11%) on the supplemented pyruvate media, while none grew on the supplemented glycerol media. This difference is likely due to the promotion of *M. bovis* growth by pyruvate and inhibition by glycerol [44].

Out of 10 colonies, only 9 were AFB staining positive and subjected to RD4 deletion. RD4 deletion typing of the 9 isolates confirmed the presence of a PCR product of size 446 bp, indicating that they were “deleted for RD4” and hence identified as *M. bovis* [18]. Furthermore, molecular characterization using spoligotyping on the nine *M. bovis* isolates revealed that 55.6% (5/9) of the isolates belonged to three different spoligotype patterns, namely, SB0134, SB2233, and SB1176, which aligns with the findings of previously reported studies conducted by Mekonnen et al. [45] and Berg et al. [46]. The most dominant spoligotypes were SB2233 and SB0134 with a prevalence of 40% (2/5). On the other hand, SB1176 was the least prevalent spoligotype with a prevalence of 20% (1/5).

The high occurrence of spoligotype SB0134 in our study aligns with the results of Mekonnen et al. [45], who reported a prevalence of 47%.

In this study, SB1176 was identified as a minor spoligotype with a frequency of 1 out of 5. Previous studies conducted by Tulu et al., [47], Biffa et al. [17], and Firdessa et al. [29] in central and other parts of Ethiopia have also reported SB1176 as the dominant spoligotype. The observed discrepancy in the results might be due to variations in geographic location, sample size, and types. It is worth noting that SB1176 has been found in several East African countries, including Ethiopia, Uganda, Burundi, and Tanzania [46].

In addition, 44.4% (4/9) of the isolates exhibited unknown patterns not registered on *M. bovis*.org; one showed a pattern similar to SB2233 but lacked spacer 9, while three other unknown isolates lacked spacers 14–16, 32–36, 39, 40, and 42.

The present study showed that crossbred cattle were found to be more susceptible to bovine tuberculosis (BTB) than local zebu breeds. This finding was consistent with results from previous studies, such as [13], which reported a 17.9% prevalence of BTB in crossbreeds and 4.4% in the local breeds, and similar findings were also reported by [18, 23, 47–49] across different Ethiopian abattoirs, which indicated that crossbreeds are more at risk for BTB clinical disease than Zebus.

The susceptibility to bovine tuberculosis (BTB) differs among different breeds of cattle due to their genetic differences. Studies such as [6] suggest that Zebu breed cattle are much more resistant to BTB than European cattle and often experience less severe symptoms. On the other hand, Yibrah [13] found that genetically enhanced breeds are far more susceptible to BTB clinical disease than local breeds. As such, it is clear that the type of cattle affects their susceptibility to BTB significantly.

In this study, sex was not a risk factor for tuberculosis lesions, consistent with studies by [11, 23, 49] from Ethiopian abattoirs. However, Shewatatek [48] found that sex was a risk factor for bovine tuberculosis in North Gondar. The difference in the variation in sex as a risk factor for bovine tuberculosis between this research and other reports may be due to the lower number of female animals slaughtered during the studied periods.

The current study found that age was not a risk factor, which agrees with the results of previous studies [11, 50]. However, the prevalence of the disease slightly increased with age. This might be because if the animal contracted the disease when it was younger, it may take some time for symptoms to show and become clinically relevant at an older age. In addition, immunosuppression potentially caused by age could also contribute to this phenomenon [6].

The results of the present study suggest that there is no statistically significant difference in the prevalence of bovine tuberculosis among groups with different body condition scores. This finding is consistent with previous research conducted elsewhere [11, 48, 49]. However, a study conducted in Gambela reported a statistically significant association between the body condition score and the prevalence of bovine tuberculosis [18]. The discrepancy between this study and others may be due to the fact that animals in poor physical condition were not slaughtered during the study period. Although the prevalence of bovine tuberculosis was slightly higher in animals with moderate physical condition (8.87%) than in those with good physical condition (2.08%), this is consistent with findings from other studies that have reported higher immunological response to infection in animals in good physical condition due to the debilitating nature of the disease [6, 40, 42].

The present findings state that the prevalence of bovine tuberculosis based on TB suspected lesions in slaughtered cattle from Nekemte Town origin (13.04%) was higher compared to those from Diga (3.97%). Previous studies by Woyessa et al. [23] reported a high rate of bovine tuberculosis lesions in cattle from Guto Gida (15.5%) and a low prevalence in cattle from Wayu tuqa. These discrepancies may be due to varying levels of infection or differences in production systems within different regions, as suggested in [14]. In this study, it was found that the highest gross tuberculosis lesions were most commonly found in the thoracic and mesenteric lymph nodes. This contradicts previous findings of other studies, which showed the retropharyngeal and submaxillary lymph nodes to be more common sites of infection. This suggests that inhalation may be the main route of infection and that postmortem examination should focus on the lungs and associated lymph nodes for comprehensive diagnosis [40, 51]. In essence, according to Corner [52], up to 95% of the cattle with visible TB symptoms could be diagnosed by examining the lungs and related lymph nodes. It was also found that infection typically occurred through ingestion when lesions were found in the mesenteric lymph nodes.

In this study, all specimens collected from presumptive tuberculosis lesions were subjected to the bacteriological culture process in order to isolate and characterize the pathogen. The results indicated that 12.5% of these specimens showed positive culture results, which were lower than the previously conducted by Alemu et al. [18] and Nemomsa et al. [53]. The low recovery rate of Mycobacterium isolates in this study could be attributed to several factors. Freezing samples or delays during transportation from collection sites can result in loss of viability of the bacteria [23]. In addition, the decontamination procedure required for the Lowenstein–Jensen culture has been reported to cause up to 60% loss of Mycobacteria [52]. The presence of caseous or calcified lesions, which may lack viable organisms, or lesions caused by nontuberculous intracellular pathogens can also lead to lack of growth in culture. Finally, misclassification of granulomatous lesions caused by organisms other than Mycobacteria during gross pathology can mistakenly include nontuberculous samples that fail to yield positive culture results [53]. Taken together, these limitations can reduce the sensitivity of bacteriological isolation from lesioned tissues.

In the current study, only 26.67% of the respondents who completed the questionnaire survey were aware of bovine tuberculosis, its zoonotic importance, and its mode of transmission. This is lower than the findings reported by Gambella town and surroundings [18], which indicated that 22% of the respondents were aware that BTB can infect cattle and 15% recognised its zoonotic potential. In addition, research in Wuchale-Jida district [54] showed that less than half (38.3%) of the participants had knowledge about BTB and its transmission from cattle to humans, which was significantly higher than the 80.7% reported in the present study. In terms of consumption habits, 14.2% of the people in this sample consumed unpasteurized milk compared to 45% in Gambella Town and surroundings [18], 52.1% in Wuchale and Jida counties [54], and 89.47% in Ambo and Toke Kutaye districts [55]. While education level impacted the habit of eating unpasteurized milk, it did not influence raw meat intake (Table 5). This result is consistent with findings from the research in Wuchale-Jida district [54]. In this study, 22.5% of the participants were aware of zoonotic TB transmission methods; however, this figure is lower than the rate observed in Wuchale-Jida County (30.8%). Moreover, most participants admitted consuming raw meat frequently, but no clear correlation between their raw meat consumption habits emerged during this study.

## 6. Conclusion and Recommendations

*Mycobacterium bovis* was confirmed as the causative agent in 9 suggestive tuberculosis lesions from 80 slaughtered cattle tissue samples using culture, acid-fast staining, RD4 deletion typing, and spoligotyping. This provides evidence that *M. bovis* is circulating and causing disease in cattle in the study area. Three distinct patterns were observed among these five isolates: SB0134, SB1176, and SB2233. In addition, 44% of the isolates were new strains not previously reported, suggesting novel *M. bovis* genotypes in the region. Crossbred cattle showed significantly higher TB positivity compared to local breeds, indicating they are at higher risk of infection. Other factors such as sex, body condition, and origin were not significantly associated with TB lesions. Knowledge regarding bovine TB was low among the public surveyed, with risky behaviors like consumption of raw milk and meat still commonly practiced. This highlights a risk of zoonotic TB transmission from cattle. These findings suggested that *M. bovis* was the main cause of bovine tuberculosis in the study area and posed a risk of disease transmission from cattle to humans due to low levels of public health awareness. As such, improved awareness among citizens and the development of control policies are recommended.

## Figures and Tables

**Table 1:** Body condition scoring of slaughtered animal.

BCS	Body fat (%)	Detailed description
1	3.77	Clearly, defined bone structure of shoulder, ribs, back, hooks, and pins easily visible. Little muscle tissue or fat present
2	7.54	Small amount of muscling in the hindquarters. Fat is present, but not abundant. Space between the spinous process is easily seen
3	11.30	Fat begins to cover loin, back, and fore ribs. Upper skeletal structures visible. Spinous process is easily identified
4	15.07	Foreribs becoming less noticeable. The transverse spinous process can be identified by palpation. Fat and muscle tissue not abundant, but increasing in fullness
5	18.89	Ribs are visible only when the animal has been shrunk. Processes not visible. Each side of the tail head is filled, but not mounded
6	22.61	Ribs not noticeable to the eye. Muscling in hindquarters plump and full. Fat around tail head and covering the foreribs
7	26.38	Spinous process can only be felt with firm pressure. Fat cover in abundance in one side of tail head
8	30.15	Animal smooth and blocky appearance; bone structure difficult to identify. Fat cover is abundant
9	33.91	Structures difficult to identify. Fat cover is excessive and mobility may be impaired

**Table 2:** Methods of determining the age of cattle.

Years	Detailed description
At birth to 1 month	Two or more of the temporary incisor teeth present. Within first month, entire 8 temporary incisors appear
2 years	As a long yearling, the central pair of temporary incisor teeth or pinchers is replaced by the permanent pinchers. At 2 years, the central permanent incisors attain full development
2 and 1/2 years	Permanent first intermediates, one on each side of the pinchers, are cut. Usually, these are fully developed at 3 years
3 and 1/2 years	The second intermediates or laterals are cut. They are on a level with the first intermediates and begin to wear at 4 years
4 and 1/2 years	The corner teeth are replaced. At 5 years, the animal usually has the full complement of incisors with the corners fully developed
5-6 years	The permanent pinchers are leveled, both pairs of intermediates are partially leveled, and the corner incisors show wear
7–10 years	At 7 or 8 years, the pinchers show noticeable wear; at 8 or 9 years, the middle pairs show noticeable wear; and at 10 years, the corner teeth show noticeable wear
12 years	After the animal passed the 6th year, the arch gradually loses its rounded contour and becomes nearly straight by the 12th year. In the meantime, the teeth gradually become triangular in shape, distinctly separated, and show progressive wearing to stubs. These conditions become more marked with increasing age

**Table 3:** The anatomical distribution of gross tuberculosis lesions in different organs and tissues.

Variables	Categories	Lymph node/organ	Number of examined cattle	Positive cattle for TB gross lesion	(%)
Anatomic site	Head	Retropharyngeal	1020	8	0.78
	Thorax	Bronchial LN	1020	17	1.7
		Mediastinal LN	1020	38	3.7
		Lung	1020	5	0.5
	Abdomen	Mesenteric LN	1020	10	1.0

Total suspected tuberculosis lesion			1020	80	7.8

**Table 4:** Exploring the associations between sex, body condition, and breed with bovine tuberculosis prevalence in cattle.

Variables	Categories	No. of tested	Positive (%)	*χ* ^2^	OR	95% CI	*P* value
Sex	Female	90	8 (8.8)	1.27	1.693	0.681–3.902	0.260
	Male	930	71 (7.65)				

BCS	Good	192	4 (2.08)	3.02	1.855	0.907–3.220	0.082
	Medium	828	73 (8.87)				

Breed	Cross	120	21 (17.6)	1.35	1.345	0.180–10.023	0.009
	Local	900	68 (7.6)				

Origin	Jima Arjo	170	11 (6.4)				0.192
	Bandra	170	15 (8.8)				
	Diga	170	6 (3.5)				
	Arjo Gudatu	170	13 (7.65)	3.2	1.96	0.917–3.620	
	Uke	170	16 (9.64)				
	Nekemte	170	22 (13.0)				

*χ* ^2^ = chi square; OR = odds ratio; CI = confidence interval.

**Table 5:** The survey results analyzed participants' knowledge, attitudes, and behaviors towards bovine tuberculosis.

KAP questions	Education	*N* = 120	Yes	(%)
Knowledge of BTB disease (causative agent, clinical sign, and morbidity and mortality rate)	Illiterate	18	1	5.5
	Primary	41	7	17.1
	Secondary	61	24	39.3
	Knowledge		32	26.7

Knowledge of zoonotic aspect TB	Illiterate	18	0	0
	Primary	41	6	14.6
	Secondary	62	22	35.5
	Knowledge		28	23.3

Habit of drinking raw milk	Primary	41	6	14.6
	Secondary	61	6	9.8
	Attitude		17	14.1

Habit of consuming raw meat	Illiterate	18	8	44.4
	Primary	41	28	68.2
	Secondary	61	46	75.4
	Attitude		82	68.3

Know tuberculosis prevention practice	Illiterate	18	0	0
	Primary	41	6	14.6
	Secondary	61	22	34.4
	Practice		27	22.5

## Data Availability

The data used to support the findings of this study are included within the article.

## Appendix

### Preparing Tissue Sample

  Homogenizing of the tissue sample
  Remove the surrounding flash with sterilized blade/scissor
  Cut the tissue into pies by using sterilized sizer
  Add these pies of tissue into a mortar
  Then, crash the tissue by pistons
  Add a small amount of sterilized saline
  Filter the granted tissue using sterilized sieve
  Harvest 5 ml of filtered liquid into the centrifuge tube
  Decontamination of the tissue
  Add equal volume of 4% NaOH to the centrifuge tube
  Mix well for approximately 10 minutes
  Centrifuge the mixed liquid at 15000 rpm at 4°C for 15 minutes
  Decant the supernatant
  Mix the sediment
  Neutralization
  A drop of phenol red indicator was added to well-mixed sediment and mixed well until it became red
  Neutralize the sediment by adding 2 N HCl dropwise until the color changes from purple to yellow.
